# Implementation of a mechanical CPR device in a physician staffed HEMS – a prospective observational study

**DOI:** 10.1186/s13049-018-0503-4

**Published:** 2018-04-28

**Authors:** Simon Rauch, Giacomo Strapazzon, Monika Brodmann, Ernst Fop, Christian Masoner, Lydia Rauch, Alessandro Forti, Urs Pietsch, Peter Mair, Hermann Brugger

**Affiliations:** 1 Institute of Mountain Emergency Medicine, EURAC Research, Viale Druso 1, 39100 Bolzano, Italy; 2 Department of Anaesthesiology, University Hospital, LMU Munich, 80337 Munich, Germany; 3 Department of Sport Science, Medical Section, University of Innsbruck, 6020 Innsbruck, Austria; 4 Department of Emergency Medicine, Inselspital, Bern University Hospital, Freiburgstrasse 16C, 3010 Bern, Switzerland; 5 Department of Prehospital Emergency Medicine (118), Via Lorenz Böhler 3, 39100 Bolzano, Italy; 6 Department of Anaesthesiology and Intensive Care Medicine, Bressanone Hospital, Via Dante 51, 39042 Bressanone, Italy; 7 Department of Anaesthesiology, Bolzano Central Hospital, Via Lorenz-Böhler 5, 39100 Bolzano, Italy; 8 Department of Anaesthesiology and Intensive Care Medicine, Treviso Hospital, Piazzale Ospedale 1, 31100 Treviso, Italy; 9 Department of Anaesthesiology, Intensive Care Medicine and Emergency Medicine, Kantonsspital St. Gallen, Rorschacherstraße 95, 9007 St. Gallen, Switzerland; 10 Department of Anaesthesiology and Intensive Care Medicine, Innsbruck Medical University Hospital, Anichstraße 35, 6020 Innsbruck, Austria

## Abstract

In this prospective, observational study we describe the incidence and characteristics of out of hospital cardiac arrest (OHCA) cases who received mechanical CPR, after the implementation of a mechanical CPR device (LUCAS 2; Physio Control, Redmond, WA, USA) in a physician staffed helicopter emergency medical service (HEMS) in South Tyrol, Italy. During the study period (06/2013–04/2016), 525 OHCA cases were registered by the dispatch centre, 271 (51.6%) were assisted by HEMS. LUCAS 2 was applied in 18 (6.6%) of all HEMS-assisted OHCA patients; ten were treated with LUCAS 2 at the scene only, and eight were transported to hospital with ongoing CPR. Two (11.1%) of the 18 patients survived long term with full neurologic recovery. In seven of eight patients transferred to hospital with ongoing CPR, CPR was ceased in the emergency room without further intervention. Retrospectively, all HEMS-assisted OHCA cases were screened for proposed indication criteria for prolonged CPR. Thirteen patients fulfilled these criteria, but only two of them were transported to hospital. Based on these results, we propose a standard operating procedure for HEMS-assisted patients with refractory OHCA in a region without hospitals with ECLS capacity.

Sir,

In this prospective observational study, we report incidence, clinical characteristics and adverse events of out-of-hospital cardiac arrest (OHCA) patients who receive mechanical CPR in the emergency medical helicopter systems (HEMS) operations in South Tyrol, Italy. The study was approved by the local ethics committee and registered in ClinicalTrials.gov (NCT01745926). In 2013, all three helicopters serving the area were equipped with the “Lund University Cardiac Arrest System 2” device (LUCAS 2; Physio Control, Redmond, WA, USA). All medical crew underwent extensive theoretical and practical training in its use. Due to the lack of evidence base and generally accepted indication criteria associated with this device, the decision to deploy LUCAS 2 in OHCA at the scene, or for ongoing CPR during transport to hospital, was left at the discretion of the emergency physician on duty. However, the following exclusion criteria for hospital transport with ongoing CPR have been defined: unwitnessed OHCA with asystole as initial rhythm and no return of spontaneous circulation (ROSC) after 20 min of ALS [1]; avalanche victim in asystolic cardiac arrest with obstructed airways and burial time > 35 min [2]; lethal injuries [3]; totally frozen body [3] and device application not possible due to anatomical limits.

During the study period (06/2013–04/2016), the following data were prospectively collected, based on the Utstein-style [4]: time and presumed cause of cardiac arrest, witnessed cardiac arrest, initial cardiac rhythm, duration of cardiac arrest and CPR, ALS interventions (i.e. defibrillation, advanced airway management, drug administration), timing of LUCAS 2 application, end-tidal CO_2_ (etCO_2_) 10–20 min after LUCAS 2 application, time to hospital arrival, patient outcome and any technical problems or adverse events encountered during LUCAS 2-CPR. At the end of the study period, we screened all HEMS-assisted OHCA cases for the indication criteria for hospital transport under continuous CPR proposed by Ortega-Deballon et al. [5]. These criteria included: witnessed cardiac arrest of non-traumatic cause, no-flow time ≤ 5 min, presence of an initial shockable rhythm, absence of severe activities-of-daily-living disability or severe co-morbidities before the cardiac arrest, and age-range of the patient between 18 and 75 years.

During the study period, a total number of 525 OHCA cases were recorded by the dispatch centre and in 271 (51.6%) HEMS was involved. In 18 (6.6%) of the HEMS assisted OHCA cases, LUCAS 2 was applied, in 10 of them, mechanical CPR was performed on scene only, whereas 8 patients were transported to hospital with ongoing mechanical CPR (Fig. 1). Patient characteristics and therapeutic interventions for these 18 patients are summarised in Table 1. All eight patients transported to hospital with ongoing CPR were admitted to the Regional Hospital of Bolzano, a tertiary care centre, where one patient had ROSC in the emergency department (ED) without further specific treatment, and survived without neurologic sequelae (cerebral performance category 1). In the remaining seven patients, CPR was ceased in the ED without further invasive in-hospital intervention. Equally, one of the 10 patients treated with LUCAS 2 at the scene survived with a good neurologic outcome. Of the 271 HEMS-assisted OHCA cases, 31 (11.4%) fulfilled the criteria for prolonged CPR proposed by Ortega-Deballon et al. [5] (Fig. 2). Of these, 18 (6.6%) patients experienced ROSC after physician provided ALS already at the scene and 13 (4.8%) remained in refractory OHCA and thus were candidates for prolonged CPR and hospital transport. Two (15%) of these 13 patients were actually transported to hospital with ongoing mechanical CPR, in the remaining 11 (85%) patients, CPR was terminated at scene by the emergency physician.

During three of the 18 applications of LUCAS 2, a dislocation of the device was reported. No other technical problems were recorded.

**Fig. 1:**
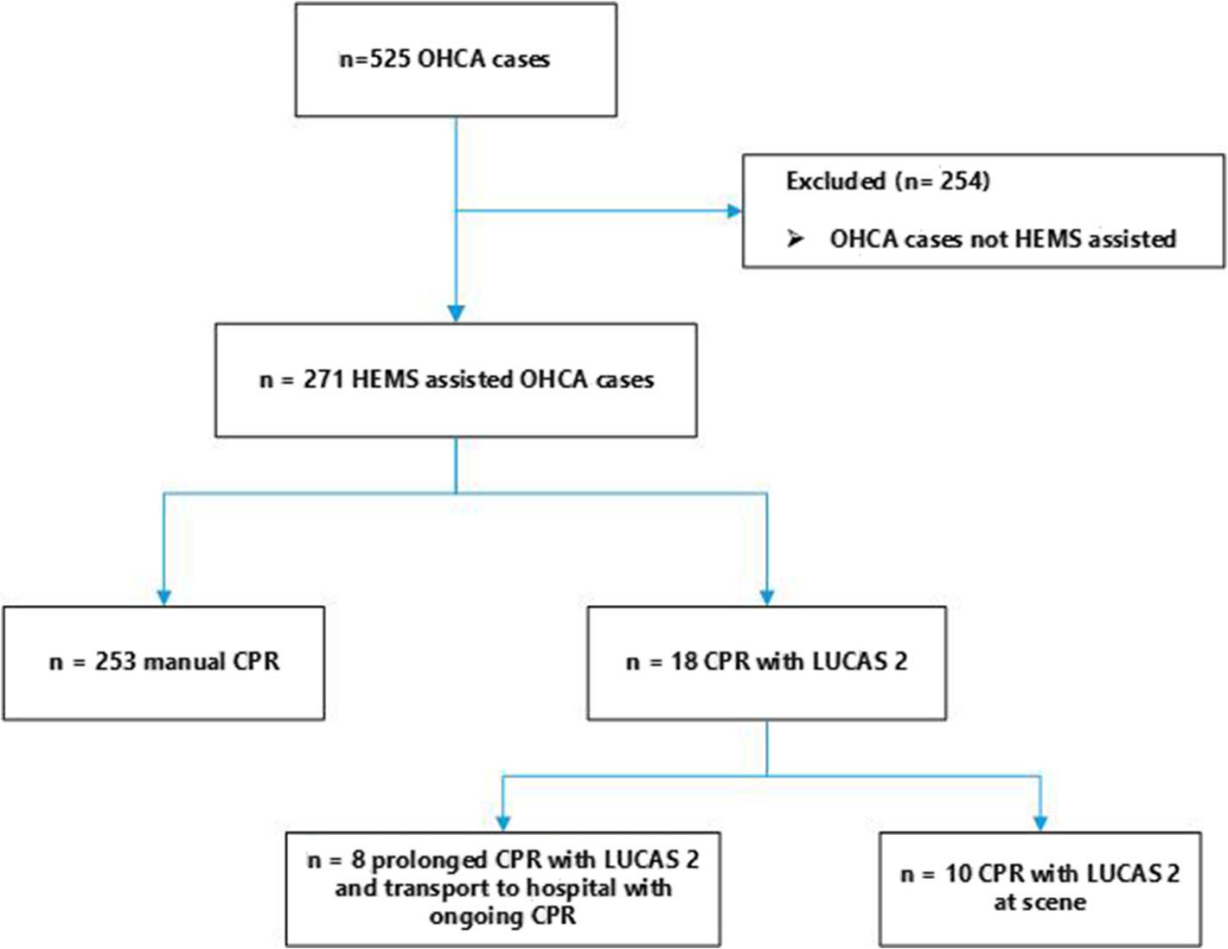
Flow chart of the study. OHCA Out-of-hospital cardiac arrest; HEMS Helicopter emergency medical system; CPR cardiopulmonary resuscitation

**Table 1:**
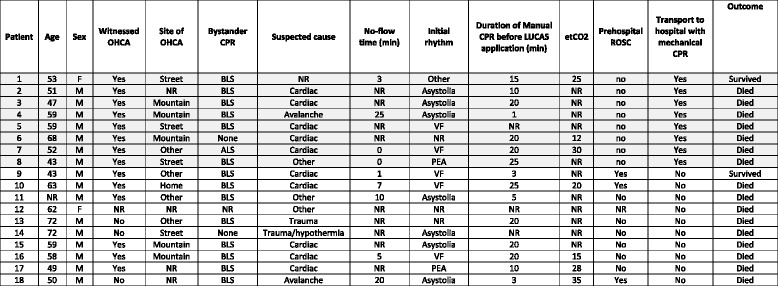
Demographics, patient characteristics, therapeutic interventions and outcome in 18 patients with HEMS assisted OHCA who received mechanical CPR

Light grey shading: patients transported to hospital with ongoing mechanical CPR. M male; F female; OHCA out-of-hospital cardiac arrest; BLS basic life support; ALS advanced life support; CPR: cardiopulmonary resuscitation; ROSC return of spontaneous circulation; etCO_2_ end-tidal CO_2_; NR not reported

**Fig. 2:**
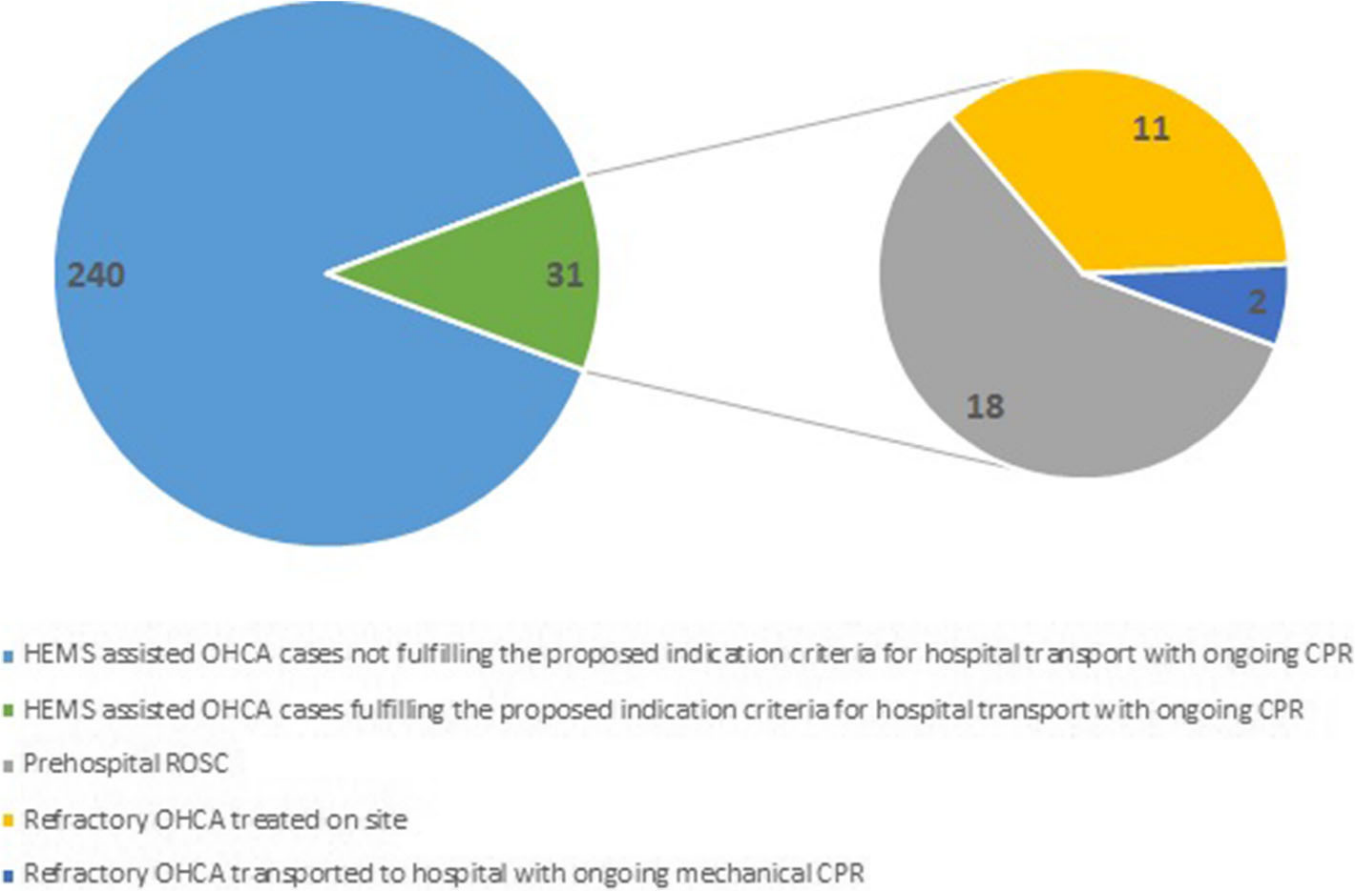
Proportion of HEMS assisted OHCA full filling criteria for hospital transport with ongoing CPR proposed by Ortega-Deballon et al. [5]

In this pragmatic observational trial with a high rate of HEMS assisted OHCA cases, LUCAS 2 was used in only 6.6% and less than half of these patients were transported to hospital with ongoing CPR in case of refractory cardiac arrest. Prolonged resuscitation efforts supported by a mechanical device are useful only in patients for whom a chance of a neurologically meaningful survival exists; thus, patient selection is of utmost importance when implementing mechanical CPR devices. In our study, only 4.8% of all HEMS-assisted OHCA cases were potential candidates for prolonged CPR and hospital transport according to the indication criteria proposed by Ortega-Deballon et al. [5]. This is slightly lower compared to recently published studies from urban areas (range 6.0–10.6%), which used similar screening criteria [6–8]. However, of the potential candidates for prolonged CPR in our study, only 15% were transported to hospital with ongoing CPR, whereas in 85% CPR was terminated at scene by the emergency physician. The reason for this finding is unclear, however, similar results were found in a study by Poppe et al. [8].

In the emergency department, CPR was ceased without further invasive interventions for all patients, except in one case with ROSC in the emergency room. This finding might be due to the fact that the majority of patients transported to hospital with ongoing CPR did not fulfil the criteria associated with a favourable prognosis after OHCA, or due the absence of a clear in-hospital operating procedure (i.e. inclusion of cardiac catheterization).

First, these findings underline the need for a standard operating procedure (SOP) including clear indication criteria before the adoption of a new technology. Second, this study demonstrates that prehospital emergency medical care should always be linked to, and analysed in, the context of hospital capabilities.

In the province of South Tyrol, no hospital has extracorporeal life support (ECLS) capacity. Yet, a large Danish study described a 30-day survival rate of 20% after refractory OHCA with ongoing CPR at hospital arrival without the use of ECLS, thus encouraging prolonged resuscitation efforts and transport to hospital in selected patients even without the use of ECLS [9]. However, internationally, the trend moves towards the deployment of ECLS in the case of refractory cardiac arrest (i.e., E-CPR) [10]. Equally, after the Australian CHEER trial [11], many centres have implemented a bundle of care for selected patients with refractory OHCA, including E-CPR, early coronary reperfusion and targeted temperature management. Data from the Extracorporeal Life Support Organisation (ELSO) report an overall survival of 29% with E-CPR in the management of refractory cardiac arrest [10].

The findings of our study have triggered a change in the prehospital management of patients with refractory cardiac arrest in South Tyrol. A SOP for HEMS assisted refractory OHCA cases was developed (Fig. 3), with distinct inclusion and exclusion criteria for prolonged CPR. These criteria were established based on the systematic review of international practices by Ortega-Deballon and co-workers [5] and in consultation with a round-table of experts from Italy, Austria and Switzerland. Furthermore, a collaboration has been established with the nearest ECLS-centres Innsbruck (Austria) and Treviso (Italy), both approximately 40 flight minutes from Bolzano (capital city of South Tyrol). Kim and co-workers found a significant increase in mortality with an E-CPR initiation > 60 min after the cardiac arrest [12]. Based on this evidence and the long transport duration to the ECLS centres, we have agreed that ALS measures with high quality CPR should be performed for a maximum of 10 min at the scene. In the absence of ROSC, the patients meeting the inclusion criteria should than be loaded into the helicopter and transported to the closest ECLS centre under ongoing mechanical CPR, in order to keep the potential low-flow time as short as possible. Education of the HEMS crews before the implementation of this SOP is of paramount importance in order to guarantee a smooth course of actions and a timely pre-announcement of the patient in the ECLS centre. We assume that this SOP could be applicable and transferrable to other rural areas with low population density, functioning HEMS but without extracorporeal life support facilities.

**Fig. 3:**
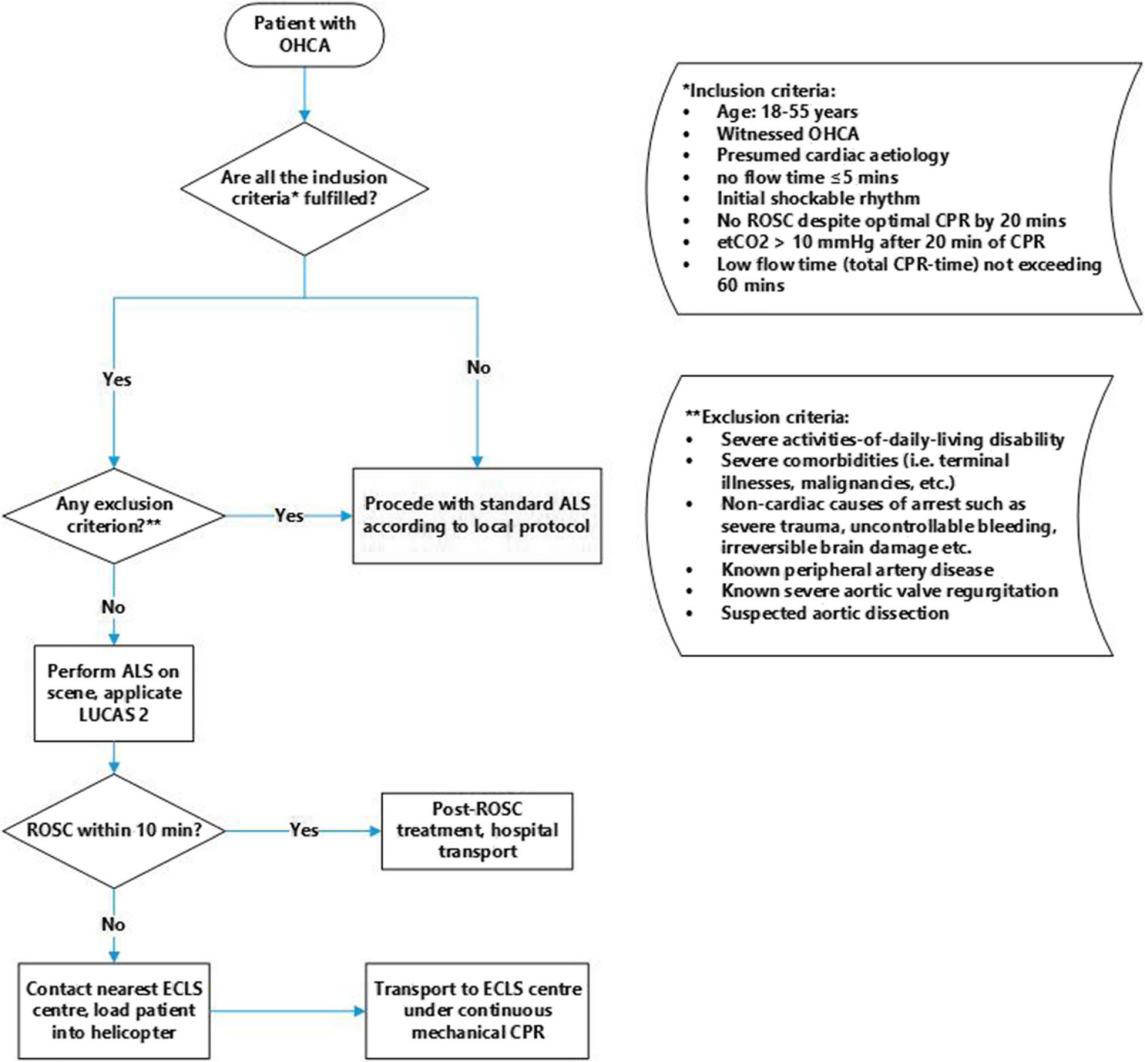
Proposed standard operating procedure for HEMS-assisted patients with refractory OHCA in a region without hospitals with ECLS capacity. OHCA out-of-hospital cardiac arrest; ALS advanced life support; ROSC return of spontaneous circulation; ECLS extracorporeal life support; etCO_2_ end-tidal CO_2_

## Abbreviations

CPR: Cardiopulmonary resuscitation
ECLS: Extracorporeal life support
E-CPR: Extracorporeal cardiopulmonary resuscitation
HEMS: Helicopter emergency medical system
OHCA: Out-of-hospital cardiac arrest
ROSC: Return of spontaneous circulation
SOP: Standard operating procedure
